# Pilot Study: Moving Towards a Scalable Intervention for Postgraduate Communication Skills Training

**DOI:** 10.1089/pmr.2024.0006

**Published:** 2024-08-01

**Authors:** Warren Lewin, Helen James, Nikolina Mizdrak, Ben Kaasa, Shira A. Strauss, J. Thomas Toguri

**Affiliations:** ^1^ Department of Family and Community Medicine, University of Toronto, Toronto, Canada.; ^2^ Division of Palliative Care, University Health Network, Toronto, Canada.; ^3^ Toronto Western Hospital Family Health Team, University Health Network, Toronto, Canada.

**Keywords:** postgraduate medical education, e-modules, simulation, advance care planning, educational design, communication

## Abstract

**Background:**

Communication skills are foundational to the practice of medicine and training to build them is recommended. Serious illness communication skills (SICSs) teaching is inconsistently and sparsely taught in postgraduate training and residents report feeling inadequately trained to have difficult conversations. The authors developed an e-module demonstrating high-yield communication skills from a known evidence-based training program to standardize core SICS teaching and questioned how using it before skills practice impacted comfort and preparedness for residents to complete advance care planning (ACP).

**Methods:**

Family medicine residents at an academic hospital in Toronto, Canada, completed a novel e-module that replaced a typical didactic-lecture introducing core SICS relevant to ACP conversations. Residents then discussed the skills, followed by practicing them deliberately in a structured role-play simulation with feedback by trained facilitators. Residents completed pre- and post-intervention attitudinal surveys.

**Results:**

Residents preferred a combination of learning modalities and welcomed online and virtual teaching methods for learning SICS. Residents reported higher levels of preparedness for engaging in ACP, delivering serious news, and discussing goals of care post-intervention. Residents showed more interest in discussing ACP post-intervention but questioned feasibility for doing so in busy ambulatory clinics.

**Conclusion:**

Scalable time-efficient teaching strategies are needed to fill a known education gap. This study demonstrated benefits of incorporating brief e-module learning into residents’ preparation for SICS training using deliberate practice simulation training. The online, interactive virtual training improved resident readiness and comfort for ACP, an area often overlooked in medical education. Moreover, it provides an evidence-informed standardized tool for clinician teachers to seamlessly incorporate into their teaching practices.

## Introduction

Communication skills are foundational to the practice of medicine with training to develop these skills being strongly recommended.^1,2^ Serious illness communication skills (SICS) such as disclosing serious news, responding to emotion, and making treatment recommendations are critical to leading high-quality advance care planning (ACP) conversations and comprise required communication competencies for family medicine residents.^3–6^ Completing ACP has myriad benefits including leading to better documentation of patients’ end-of-life (EOL) wishes, improved quality of EOL care, and increased patient/family satisfaction of care received.^7^ Yet, SICS teaching is inconsistently and sparsely taught in postgraduate training,^2,8^ and residents report feeling inadequately trained to have difficult conversations.^2^ When taught, common postgraduate communication teaching methodologies include observation, didactic lectures, and simulation.^8^ These teaching methods are dependent on faculty and learners devoting time synchronously^8^ and risk variation in faculty knowledge and teaching proficiency that can lead to undesirable inconsistencies in student learning.

Simulation training from a well-known SICS training organization, VitalTalk (vitaltalk.org), has been used at one of the training sites at our institution since 2018. This evidence informed training program, taught by faculty that completed VitalTalk’s train-the-trainer course to strengthen and standardize local teaching delivery, provides learners and faculty with a structured and replicable approach to developing SICS supporting high-quality ACP.^9^

At present, family medicine trainees at one site engage annually in a three-hour workshop, powered by VitalTalk, to learn about and practice SICS guided by the approach described in Childers.^10^ The first hour comprises an interactive lecture introducing skills such as sharing serious health information and responding to emotion. The remainder of the workshop focuses on small group sessions dedicated to deliberate practice of SICS with simulated patients using cases tailored specifically for family medicine and have been locally developed.

At our institution, family medicine residents are assigned to train at one of 14 distributed sites, each with its own unique approach to teaching a core curriculum. This results in variability in SICS instruction, if provided at all. We completed a local needs assessment that identified family medicine trainee preferences to learn about SICS asynchronously and before attending simulation training. To respond to these needs, we developed an e-module to standardize the SICS taught in a “powered by VitalTalk workshop”, made it accessible online, and piloted its use during one of our routine workshops. The aim was to test its feasibility with family medicine residents, determine its impact on residents’ preparedness for ACP, and inform potential scaling across the training sites.

## Methods

**FIG. 1.**
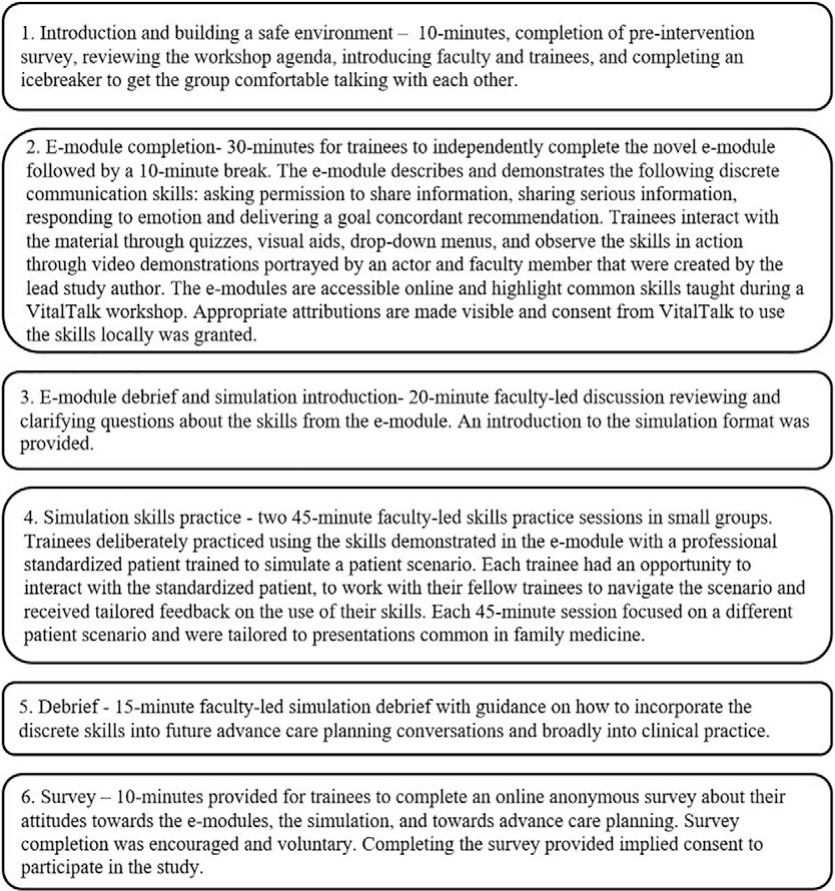
Outline of structure for the residents’ 3-hour workshop.

First and second-year family medicine residents at an academic hospital in Toronto, Canada participated in one three-hour virtual SICS workshop powered by VitalTalk. The workshop was integrated into the trainees’ protected teaching schedule and comprised e-module, discussion, and simulation activities. The e-module was created by the lead study author. The workshop structure is outlined in Figure 1.

Residents completed voluntary and anonymous surveys requesting demographic information and assessing their perceived preparedness for ACP discussions, as well as their attitudes toward ACP training and practice. Surveys were completed online at the beginning and end of the workshop (Appendix 1). We could not find a validated survey that met our criteria, and so the authors developed one for this project.

Survey responses from the Likert scales were recoded to binary positive (>5) and neutral or negative (≤4) then analyzed with McNemar’s test for dependent proportions with Edward’s correction.

Research ethics board exemption was provided by our institution. (QI ID #22–0496).

## Results

### Demographics

A total of 16 out of 23 (70%) eligible family medicine residents participated in the workshop. Fifteen (94%) were included in the analysis due to an incomplete post-survey from one respondent. Baseline characteristics are reported in Table 1.

**Table 1.** Family Medicine Resident Participant Demographics

	Total (%)
Postgraduate year 1	10 (66.7)
Postgraduate year 2	5 (33.3)
Male	8 (53.3)
Female	7 (46.7)
Year of birth, mean	1994 (range 1989–1997)
Location of medical school training	
** **Canada	13 (86.7)
** **Poland	1 (6.7)
** **The Caribbean	1 (6.7)

### Preparedness

Residents’ perceived preparedness towards ACP shows improvement consistently across questions, although not statistically significant (Table 2). Residents felt more prepared to engage in ACP discussions (pre = 0%, post = 0.13%, *p* value = 0.48), to deliver serious news (pre = 0.13%, post = 0.2%, *p* value = 1), to elicit a patient’s goals of care (pre = 0%, post = 0.13%, *p* value = 0.48), and to discuss treatment options (pre = 0.067%, post = 0.13%, *p* value = 1).

**Table 2.** Resident Reported Level of Preparedness towards Key ACP Topics before and after Training

Question	Number of positive responses in pre-survey *n* (%)	Number of positive responses in post-survey *n* (%)	McNemar’s *p* value (with Edward’s correction)
How well prepared do you feel to overall engage in advanced care planning (ACP) conversations, a process in which a patient reflects upon their goals, values and beliefs, allowing them to make decisions about their future medical treatments that align with their goals and values, with patients/family?	0 (0)	2 (0.13)	0.48
How well prepared do you feel to give bad news to a patient/family about their loved one’s illness?	2 (0.13)	3 (0.2)	1
How well prepared do you feel to elicit a patient’s goals-of-care, a treatment plan that aligns and reflects the patients’ values and what are important to them?	0 (0)	2 (0.13)	0.48
How well prepared do you feel to discuss treatment options, including comfort care, with seriously ill patients and/or their families?	1 (0.067)	2 (0.13)	1
How well prepared do you feel to overall engage in ACP conversations with patients/family?	0 (0)	4 (0.27)	0.134

### Attitudes towards the training

Over half of the residents preferred a combination of teaching methods (*n* = 8, 53%) including e-module, discussion, and simulation, over any single educational modality alone for ACP learning. Over half the residents (*n* = 9, 60%) preferred to learn pre-simulation knowledge through an e-module instead of a didactic lecture and most agreed the e-module (*n* = 13, 87%) and simulation (*n* = 13, 87%) was beneficial to their learning. Only 4/15 residents provided written feedback and no themes identified.

### Attitudes towards ACP

Post-intervention, residents reported a higher level of agreement with the following statements: “I have had sufficient practice with discussing ACP” (pre = 0.27%, post = 0.33%, *p* value = 1), and “My residency program has provided sufficient education on ACP” (pre = 0.2%, post = 0.53%, *p* value = 0.074). The training significantly increased their willingness to initiate ACP conversations (pre = 0.47%, post = 0.6%, *p* value = 0.48), but there was minimal change to residents’ views that ACP is feasible during routine clinic visits (pre = 0.2%, post = 0.33%, *p* value = 0.25). (Table 3).

**Table 3.** Residents’ Perceived Level of Agreement towards ACP and Residency Training Pre and Post Intervention

Question	Number of positive responses in pre-survey i (%)	Number of positive responses in post-survey *n* (%)	McNemar’s *p* value (with Edward’s correction)
Please rank your agreement with the statements below: I have had sufficient practice with discussing advance care planning.	4 (0.27)	5 (0.33)	1
Please rank your agreement with the statements below: My residency program has provided sufficient education on advance care planning.	3 (0.2)	8 (0.53)	0.074
Please rank your agreement with the statements below: I am interested in talking to my patients about advance care planning.	7 (0.47)	9 (0.6)	0.48
Please rank your agreement with the statements below: Advance directives should only be discussed with patients with chronic conditions.	2 (0.13)	3 (0.2)	1
Please rank your agreement with the statements below: Advance care planning should be routinely incorporated into primary care.	10 (0.67)	11 (0.73)	1
Please rank your agreement with the statements below: Discussing advance care planning during a routine clinic visit is feasible.	2 (0.13)	5 (0.33)	0.25

## Discussion

This Canadian study, focusing on a virtual VitalTalk-powered SICS workshop for family medicine residents, showcased the effectiveness of incorporating both e-module and simulation learning. Introducing practical, evidence-based SICS through an e-module followed by deliberate practice with observed feedback resulted in an increase in family medicine residents’ perceived preparedness to engage in ACP.

Previous studies using VitalTalk-powered workshops have shown improvement in the same direction in preparedness to use skills, however necessitated more time to complete.^11,12^ The ever-increasing demands on both learner and faculty time, combined with a palliative care workforce shortage,^13^ makes it imperative to find scalable, resource-efficient methods of teaching this important competency.

This study demonstrated that incorporating e-module learning into a known three-hour virtual SICS workshop is feasible. The e-module may also be used independently from a workshop and standardizes the evidence-based teaching of foundational SICS for sites. Once learners are given access to the e-modules, they can be reviewed online at any time and from anywhere. Should the e-modules prove to be an equally effective way of teaching learners these concepts, this will open up new possibilities of aligning instruction with best practices in education, such as distributed practice.^14^ In addition to being beneficial for learners, having access to a standardized e-module enables faculty to have a clear standard they can teach from and evaluate learners against, as well as incorporate into their curriculum. Furthermore, we postulate that the e-module may also have benefits to skill building, honing practice, and teaching acumen for faculty unable to access or devote the time to continuing medical education courses on this topic through observing the skills and structure in the e-modules.

Residents welcomed the multimodal teaching methods used in this study. The novel 30-minute e-module replaced a didactic lecture and made time for facilitated discussion, which provides residents with the opportunity to learn through social learning with an experienced facilitator and peers. Moreover, additional time-savings may extend to faculty interested in incorporating the already-prepared e-module into their teaching. Finally, asynchronous pre-workshop completion of the e-module could add further benefit by affording more time for deliberate skills practice in simulation.

Residents in this study felt more prepared to engage in ACP but did not agree that ACP was feasible in a routine family clinic visit, even after completing the training. This may be owing to the perceived time required for fulsome and iterative ACP conversations that may not be conducive to the busy family medicine clinic environment. Future studies are needed to assess how best to train clinicians to practically deliver ACP in the clinic setting.

## Limitations

Our study has several limitations, including the small sample size from a single institution, which may impact generalizability of the findings, as well as reliance on unvalidated self-assessment metrics. Additionally, this study did not include follow-up measures to determine if preparedness towards ACP was maintained over time or if it translates into skill competency in practice. Future research is needed to determine the type and amount of SICS training needed to support longitudinal behavior change, and importantly if integrating SICS into practice is associated with good patient-reported experience measures^15^ related to ACP and subsequent goal-concordant care.

Additionally, many postgraduate programs may be unable to add to their existing busy curricula or may prioritize other competencies^5^ in their curriculum. At our institution, core protected teaching across sites occurs weekly for three hours, and therefore, this workshop has the potential to be scaled across at training sites. This would either require investment in faculty development or utilizing an already-trained cohort of teaching faculty to virtually deliver the programming across sites, which may be cost-saving.

## Conclusion

Effective communication is an essential competency for practicing clinicians and plays a pivotal role in delivering serious illness care. This pilot study described a novel and scalable SICS online teaching tool that, combined with simulated role-play, enhanced resident knowledge and preparedness towards ACP. Next steps include evaluating the asynchronous use of the e-module before the workshop and assessing faculty attitudes toward incorporating the e-module into curriculum, aiming to expedite the closure of the communication skills teaching gap.

## Appendix

### Pre- and Post-Training Session Survey Questions excluding Demographic Questions

Improving Family Medicine Residents’ Goals of Care Discussions Through Online Based e-module and Simulation

**1. How prepared do you feel to do the following:**

**Table:**

	Not prepared, do not know the general principles/skills involved in the discussions	Not prepared to lead a discussion by myself, but aware of the general principles/skills	Somewhat prepared, know the basics, and have trouble starting and moving conversations forward	Moderately prepared, I can start a discussion but feel that I get ‘‘stuck’’ easily	Adequately prepared, but I have discomfort during challenging situations	Pretty well prepared, although I can benefit from additional experience	Extremely well prepared, at an ‘‘expert’’ level, and I can teach others
Overall engage in Advance Care Planning* conversations	1	2	3	4	5	6	7
Give bad news to a patient/family about their loved one’s illness	1	2	3	4	5	6	7
Elicit a patient’s goals-of-care, a treatment plan that aligns and reflects the patients’ values and what are important to them	1	2	3	4	5	6	7
Discuss treatment options, Including comfort care, with seriously ill patients and/or their families	1	2	3	4	5	6	7

*A process in which a patient reflects upon their goals, values and beliefs, allowing them to make decisions about their future medical treatments that align with their goals and values, with patients/family.

**2. How much do you agree or disagree with the following statements:**

**Table:**

	Strongly disagree	Disagree	Somewhat disagree	Neither agree or Disagree	Somewhat agree	Agree	Strongly agree
I have had sufficient practice with discussing advance care planning	1	2	3	4	5	6	7
My residency program has provided sufficient education on advance care planning	1	2	3	4	5	6	7
I am interested in talking to my patients about advance care planning	1	2	3	4	5	6	7
Advance care planning should be routinely incorporated into primary care	1	2	3	4	5	6	7
Discussing advance care planning during a routine clinic visit is feasible	1	2	3	4	5	6	7

**3. Is there anything you would add or take away from the modules (leave blank if not yet completed)?** *Free-text response*

**4. Which educational modality did you find most useful (leave blank if e-module/simulation not yet completed)?**

- E-module
- Didactic
- Simulation (e.g. with standardized patients)
- Combination of the above

**5. Do you prefer an e-module introduction to this type of session over a didactic one? (leave blank if modules and simulation not yet completed)**

- Yes
- No
- Other (*Free-text response*)

**6. Please rank your agreement with the statements below (leave blank if modules and simulation not yet completed):**

(a) **The e-module was beneficial to my learning**
(b) **The simulation was beneficial to my learning**

*Please answer based on the following scale:*

- Strongly disagree
- Disagree
- Somewhat disagree
- Neither agree or disagree
- Somewhat agree
- Agree
- Strongly agree

**7. Are there other additional palliative care topics you think should be discussed or are not addressed in our training?** *Free-text response*

## Abbreviations Used

ACP: advance care planning
GOC: goals of care
SICS: Serious illness communication skills

## References

[1] Daubman B, Bernacki R, Stoltenberg M, et al. Best practices for teaching clinicians to use a serious illness conversation guide. Palliative Med Reports 2020:135–142; doi: 10.1089/pmr.2020.0066 PMC8241361 34223467

[2] Nakagawa S, Fischkoff K, Berlin A, et al. Communication skills training for general surgery residents. J Surg Educ 2019;76(5):1223–1230. 31005480 10.1016/j.jsurg.2019.04.001

[3] Frank JR, Snell L, Sharbino J, eds. CanMEDS 2015 Physician Competency Framework. Royal College of Physicians and Surgeons of Canada: Ottawa; 2015.

[4] Blacker S, Downar J, Hirst L, et al. Ontario Palliative Care Network: Competency Framework. Ontario Palliative Care Network; 2019.

[5] Shaw E, Oandasan I, Fowler N, eds. CanMEDS-FM 2017: A competency framework for family physicians across the continuum. The College of Family Physicians of Canada: Mississauga, ON; 2017.

[6] Exploring the ACGME Core Competencies. NEJM Knowledge; 2016.

[7] Detering KM, Hancock AD, Reade MC, Silvester W. The impact of advance care planning on end-of-life care in elderly patients: Randomised controlled trial. BMJ 2010;340:c1345. 20332506 10.1136/bmj.c1345 PMC2844949

[8] Tan XH, Foo MA, Lim SLH, et al. Teaching and assess communication skills in the postgraduate medical setting: A systematic scoping review. BMC Med Educ 2021;21(1):483. 34503497 10.1186/s12909-021-02892-5 PMC8431930

[9] Bays AM, Engelber RA, Back AL, et al. Interprofessional communication skills training for serious illness: Evaluation of a small-group, simulated patient intervention. Journal of Palliat Med 2014;17(x):159–166. 24180700 10.1089/jpm.2013.0318 PMC4047839

[10] Childers JW, Back AL, Tulsky JA, Arnold RM. REMAP: A framework for goals of care conversations. J Oncol Pract 2017;13(10):e844–e850. 28445100 10.1200/JOP.2016.018796

[11] Lockwood BJ, Gustin J, Verbeck N, et al. Training to promote empathic communication in graduate medical education: A shared learning intervention in Internal Medicine and General Surgery. Palliat Med Rep 2022;3(1):26–35. 35415720 10.1089/pmr.2021.0036 PMC8994435

[12] Uemura T, Ito K, Yuasa M, et al. Enduring positive impact of a virtual communication skills workshop of VitalTalk pedagogy in a Non-U.S. Setting. American Journal of Hospice & Palliative Medicine 2024;41(4):424–430. 37216960 10.1177/10499091231177863 PMC11267240

[13] Vogel L. Canada needs twice as many palliative specialists. CMAJ 2017;189(1):E34–E35; doi: 10.1503/cmaj.109-5354 28246259 PMC5224958

[14] Benjamin AS, Tullis J. What makes distributed practice effective? Cogn Psychol 2010;61(3):228–247; doi: 10.1016/j.cogpsych.2010.05.004 20580350 PMC2930147

[15] Canadian Institute for Health Information. Data Quality Documentation for Users: Canadian Patient Experiences Reporting System. Ottawa, ON: CIHI; 2022.

